# Natural Zeitgebers Under Temperate Conditions Cannot Compensate for the Loss of a Functional Circadian Clock in Timing of a Vital Behavior in *Drosophila*

**DOI:** 10.1177/0748730421998112

**Published:** 2021-03-22

**Authors:** Franziska Ruf, Oliver Mitesser, Simon Tii Mungwa, Melanie Horn, Dirk Rieger, Thomas Hovestadt, Christian Wegener

**Affiliations:** * Neurobiology and Genetics, Würzburg Insect Research, Theodor-Boveri-Institute, Biocenter, University of Würzburg, Würzburg, Germany; † Animal Ecology and Tropical Biology, Theoretical Evolutionary Ecology Group, Theodor-Boveri-Institute, Biocenter, University of Würzburg, Würzburg, Germany

**Keywords:** adaptive behavior, clock plasticity, circadian dominance, PDF signaling, natural conditions, eclosion, behavioral rhythms

## Abstract

The adaptive significance of adjusting behavioral activities to the right time of the day seems obvious. Laboratory studies implicated an important role of circadian clocks in behavioral timing and rhythmicity. Yet, recent studies on clock-mutant animals questioned this importance under more naturalistic settings, as various clock mutants showed nearly normal diel activity rhythms under seminatural zeitgeber conditions. We here report evidence that proper timing of eclosion, a vital behavior of the fruit fly *Drosophila melanogaster*, requires a functional molecular clock under quasi-natural conditions. In contrast to wild-type flies, *period^01^* mutants with a defective molecular clock showed impaired rhythmicity and gating in a temperate environment even in the presence of a full complement of abiotic zeitgebers. Although *period^01^* mutants still eclosed during a certain time window during the day, this time window was much broader and loosely defined, and rhythmicity was lower or lost as classified by various statistical measures. Moreover, peak eclosion time became more susceptible to variable day-to-day changes of light. In contrast, flies with impaired peptidergic interclock signaling (*Pdf^01^* and *han^5304^* PDF receptor mutants) eclosed mostly rhythmically with normal gate sizes, similar to wild-type controls. Our results suggest that the presence of natural zeitgebers is not sufficient, and a functional molecular clock is required to induce stable temporal eclosion patterns in flies under temperate conditions with considerable day-to-day variation in light intensity and temperature. Temperate zeitgebers are, however, sufficient to functionally rescue a loss of PDF-mediated clock-internal and -output signaling

Endogenous timing via circadian clocks confers adaptive advantages as it allows organisms to anticipate daily changes in the environment (Krittika and Yadav, 2019; Vaze and Sharma, 2013; Yerushalmi and Green, 2009). In terms of behavior, the fitness relevance of being able to schedule locomotor activity, feeding, mating, or other actions at the right time of the day is intuitive as it may help maximize success and reduce risks. Many studies under constant laboratory conditions have revealed a key role of central and peripheral clocks in behavioral timing across animals. However, the importance of circadian clocks in daily timing of behavior under natural conditions or in ecological context has come under debate, as studies in the last decade have assessed the functional importance of endogenous clocks under (semi)natural conditions in a variety of mostly vertebrate species (Helm et al., 2017; Vaze and Sharma, 2013; Yerushalmi and Green, 2009). One important conclusion derived from these studies is that diel activity rhythms can remarkably differ between seminatural and laboratory conditions, because the phase relationship between behavioral activity and a given zeitgeber such as light is modulated by other abiotic zeitgebers, particularly temperature. Furthermore, intraspecific and interspecific interactions such as predation (Fenn and Macdonald, 1995; Gattermann et al., 2008; Kitchen et al., 2000) or competition for food (van der Veen et al., 2006) determine (“mask”) activity patterns in the wild. Under seminatural conditions in an outdoor enclosure, *Per2^BRDM1^* mice carrying a mutation in a core clock gene showed a very similar activity pattern as controls, and both showed mostly diurnal feeding although they are strongly nocturnal under laboratory conditions (Daan et al., 2011). In the wild, chipmunks with a lesion in the suprachiasmatic nucleus (SCN, the “master clock” in mammals) showed above-ground activity patterns similar to controls, although SCN-lesioned animals exhibit more night-time activity inside the den (DeCoursey and Krulas, 1998; DeCoursey et al., 2000). In case of voles and larger mammals, feeding and energy metabolism appear to be key drivers of diel activity patterns (Hazlerigg and Tyler, 2019; van der Veen et al., 2006) that mask circadian control. Together, these findings appear to challenge “circadian dominance” (Hazlerigg and Tyler, 2019), and instead attribute more importance to external and internal zeitgebers in the daily timing of behavior in nature.

The fruit fly (*Drosophila melanogaster*) is the best studied invertebrate model in circadian research. *Drosophila* shows robust behavioral rhythms in the laboratory, including locomotor activity. Under normal light:dark conditions (LD), *Drosophila* has a biphasic locomotor activity pattern with a morning (M) and evening (E) activity peak around lights-on and lights-off (Grima et al., 2004; Hamblen-Coyle et al., 1992). This pattern is maintained in constant darkness (DD) and has been highly reproducible across laboratories. Hence, it was surprising when the first study using activity monitors placed outdoors reported that fruit flies behave quite differently under quasi-natural conditions, becoming more diurnal than crepuscular (Vanin et al., 2012). At higher temperatures, the flies exhibited increased midday locomotor activity (A peak) instead of the siesta phase seen in the laboratory even at same temperatures. While demonstrating strongly impaired rhythmicity under LD and arrhythmicity under constant conditions in the laboratory (e.g., Hamblen-Coyle et al., 1992; Helfrich-Förster, 2001), clock-impaired flies showed a high degree of locomotor rhythmicity with little difference in activity patterns compared with wild-type controls (Vanin et al., 2012), reminiscent of the results for *Per2^BRDM1^* mice (Daan et al., 2011). The onset of morning activity in wild-type and clock-impaired flies was inversely related to night temperature and hence seems to represent a temperature response rather than a clock-controlled activity. The onset of the evening activity peak was in contrast clock-dependent at lower temperatures, with a strong temperature modulation at higher temperatures (Vanin et al., 2012). In the laboratory, nature-like simulated twilight regime is sufficient to induce wild-type-like locomotor rhythmicity and activity patterns with M and E peak activity in *per^*01*^* and *tim^01^* clock mutants even at constant temperature (Schlichting et al., 2015), providing further evidence for a subordinate role of the circadian clock in controlling the daily locomotor activity pattern. While these studies question the “circadian dominance” of daily activity patterns, they all are restricted to locomotor activity—a component common to many ecologically important behaviors in nature, from foraging and social interactions to escape from predators or adverse conditions. Locomotor activity is thus a behavior prone to a variety of environmental and endogenous signals, and the clock-driven locomotor activity rhythms are sensitive to masking.

We therefore hypothesized that “circadian dominance” with all its intuitive and likely advantages (Helm et al., 2017; Krittika and Yadav, 2019; Vaze and Sharma, 2013) may become more evident in other behaviors, especially those that serve only one particular function. Eclosion, the emergence of the adult holometabolic insect from the pupa, is arguably one of the most specific behaviors in insects, with only one dedicated function (propelling the pharate adult out of the pupal case). Occurring only once in a lifetime, eclosion in *Drosophila* is a rhythmic event on the population level gated to dawn by the interaction of the central clock in the brain and a peripheral clock in the prothoracic gland (Myers et al., 2003; Pittendrigh and Bruce, 1959; Selcho et al., 2017). Moreover, once initiated, eclosion behavior cannot be interrupted and stereotypically follows a fixed action pattern (Gammie and Truman, 1997; Kim et al., 2006), a condition under which internal timing might become especially important. Unlike most other behaviors, eclosion is largely free of motivational states and interindividual interactions; flies start to feed, mate, and interact only several hours after eclosion. Eclosion rhythmicity of *Drosophila* can be entrained by light and temperature changes (Konopka, 1972), and once entrained, rhythmicity is stable under constant conditions even if the fly population was synchronized by only a brief light pulse or temperature step during larval development (Konopka, 1972; Pittendrigh and Minis, 1964; Zimmerman et al., 1968). Flies with a mutation in the core clock genes *period* (*per*) or *timeless* (*tim*) eclose arrhythmically under constant conditions and also under laboratory LD cycles (De et al., 2012; Konopka, 1972; Konopka and Benzer, 1971; Qiu and Hardin, 1996; Sehgal et al., 1994), suggesting a general requirement of a functioning molecular clockwork for eclosion rhythmicity. In fact, the identification of the original *per^01^* (Konopka and Benzer, 1971) as well as *tim^01^* (Sehgal et al., 1994) alleles was based on genetic screens for altered eclosion rhythms. Under seminatural conditions in the tropical zone, however, a significant larger portion of *per*^0^** flies eclosed during the time around dawn compared with laboratory LD conditions (De et al., 2012).

We here report results on the eclosion rhythmicity of wild-type and clock-related mutant flies under quasi-natural temperate conditions using an open eclosion monitor (Ruf et al., 2017) which allows direct contact of the pupae with the ambient air. Compared to wild-type controls, eclosion rhythmicity was significantly altered in *per*^*^01^*^ clock mutants but not in flies with a loss of peptide-mediated phasing between central clock neuron groups. Our results argue that a functional molecular clock is required for robust normal eclosion rhythmicity under environmental conditions with frequent and pronounced day-to-day variations in light intensity and temperature.

## Materials and Methods

### Flies

For the eclosion experiments, the following fly strains were used: wild-type Canton S (WT_CS_), *w*^+^*per*^01^**, *w*^+^*per*^*^01^*^; *tim^01^*, *w*^+^*han*^*5304*^(Hyun et al., 2005),and *y w*^+^;;*Pdf^01^* (Renn et al., 1999) (kind gifts of Charlotte Förster). WT_CS_ and per^01^ originated from the Kyriacou lab (Leicester) (Vanin et al., 2012). The *Pdf^01^* line had been cantonized by Taishi Yoshii a few years ago; the genetic backgrounds of the other mutants are not equivalent. Flies were kept on standard food.

### Eclosion Under Laboratory Conditions

Flies were raised under 12 h light, 12 h dark (LD12:12) regime at 20 °C and 65% humidity (light entrainment), or at constant red light (λ = 635 nm) and 65% humidity with 12 h at 25 °C, 12 h at 16 °C (WC12:12, temperature entrainment with temperature ramped by 0.1 °C/min between conditions), or at constant red light (λ = 635 nm) at 20 °C and 12 h at 70% humidity, 12 h at 30% relative humidity (RH) (HD70:30; humidity entrainment). Eclosion was monitored for 1 week at 65% humidity either under LD12:12 or DD using TriKinetics eclosion monitors (Waltham, MA, USA) at 20 °C, or under infrared light (λ = 850 nm) under WC12:12 or constant 16 °C (CC) by the open WEclMon system with puparia mounted as previously described (Ruf et al., 2017). The results from both monitoring systems as well as constant red or infrared illumination in the WEclMon are fully and mutually comparable (Ruf et al., 2017).

To assess the effects of RH, flies were allowed to lay eggs overnight on standard food. Afterward, adult flies were removed and the eggs/larvae were kept in a climate chamber (25 °C; 60% ± 2% RH) under LD12:12. After pupariation, the food source was removed and puparia were transferred to an incubator set at different RH values (2%, 60%, or 80%) at LD12:12, 25 °C, 1 or 2 days before eclosion. Light regime and temperature were kept the same as during development. After a few days, eclosed flies and unopened puparia were counted, and eclosed flies were checked for successful wing expansion.

### Eclosion Under Natural Conditions

Experiments under natural conditions were conducted from July to October 2014 and July to September 2016 in a shelter shaded by bushes at the bee station/Hubland campus of the University of Würzburg (49°47′N, 9°56′E) (Ruf et al., 2017). During this period, the maximum temperature during the successful experiments reached 35.4 °C (20 July 2014), the lowest temperature 7.6 °C (28 September 2014), and the photoperiod varied from 1550 h (nautical dawn 0351-2259 h) to 1136 h (nautical dawn 0611-2006 h). The shelter was roofed and open at 3 sides. To prevent flies from escaping to the environment, the open sides were stretched with air-permeable black gauze. Double-sided sticky tape was glued around each monitor to trap predators and freshly eclosed flies. Flies were continuously bred inside the shelter as described (Ruf et al., 2017). Once most of the larvae had pupariated, vials were transferred to the laboratory and puparia were collected and mounted as described above during the photophase at room temperature; the whole procedure took between 3 and 4 h. Populated eclosion plates were then directly placed back into the shelter and eclosion was monitored for 1 week under constant red (λ_max_ = 635 nm) or infrared (λ_max_ = 850 nm) light using WEclMon. Eclosion rhythmicity was similar under red or infrared illumination (Ruf et al., 2017). Light intensity, temperature, and RH were registered with a datalogger (145S; MSR Electronics GmbH, Seuzach, Switzerland), placed directly at the side of the monitors.

### Data Analysis

Data were continuously obtained every 10 min (WEclMon) or every minute (TriKinetics DEM) and binned into 1-h bins for analysis. As eclosion rhythmicity occurs on the population level and depends on development, it can be recorded for 4-5 cycles only. As there is no ideal analysis method for this kind of data, we used a set of standard methods in rhythm research (Leise, 2017) based on different mathematical approaches and assumptions. Rhythmicity of the eclosion profiles was analyzed by autocorrelation implemented in a MATLAB (MathWorks, Inc., Natick, MA, USA) toolbox developed by Levine, Dowse, and colleagues (Levine et al., 2002); by a Fourier-based Lomb-Scargle (LS) analysis using ActogramJ (Schmid et al., 2011); and by cosinor model fitting to a 24-h period and subsequent zero-amplitude test using the web-based application “Cosinor.Online” (Molcan, 2019). For autocorrelation, rhythmicity was assessed by the rhythmicity index (RI, essentially the height of the third peak in the correlogram). The RI is dimensionless, is quite robust against distortions by low numbers (inevitable during eclosion monitoring for days 4-5), and is a measure of the long-range regularity of rhythmicity as well as its robustness (Levine et al., 2002). It does not depend on the amplitude or type of waveform of the oscillation (Leise, 2017). We deem the RI especially useful as it takes changes in the waveform (i.e., gate width) into account, and use the standard classification: RI > 0.3 = strong rhythmicity, 0.1 < RI < 0.3 = weak rhythmicity, and RI < 0.1 = arrhythmicity. For LS, we calculated the nonlinear power (value above the 5% significance level which indicates rhythmicity strength). The LS test is better suited to test for significant rhythmicity than autocorrelation, but assumes a sinusoidal waveform and works best with longer time series (Leise, 2017; Refinetti, 2016). The cosinor method fits a cosine wave and can thus be applied to data as short as one period, but is quite simplistic (Refinetti, 2016). For the outdoor experiments, we additionally assessed the degree of rhythmicity and gate width by Winfree’s *R* (Winfree, 1970). First, we identified the 8-h period of the day that contained the highest number of eclosion, and *R* was calculated as the number of eclosions outside this 8-h gate, divided by the number of eclosions within this gate, multiplied by 100. *R* < 60 represents strong eclosion rhythmicity, 60 < *R* < 90 represents weak rhythmicity, and *R* > 90 represents arrhythmic eclosion (Goto et al., 2006).

Statistical analysis with one-way analysis of variance (ANOVA) followed by Tukey’s post hoc test and independent-samples *t* test (for parametric data) or Kruskal-Wallis test followed by Wilcoxon paired tests (for nonparametric data) was performed in R (version 3.2.0; https://www.r-project.org/). Circular-linear correlation and circular statistics (mean, vector length, standard deviation) were analyzed and plotted using Oriana 4.02 (Kovach Computing Services, Pentraeth, Isle of Anglesey, UK). Graphs of outdoor eclosion profiles were compiled in R.

### Logistic Regression Model

We analyzed the data for day-time of eclosion using a logistic regression approach in R with focus on the timing of eclosion within a day, whereas the date of emergence was treated as random factor. RH and temperature were strongly correlated (*R* = 0.9, *p* < 0.001), and we excluded humidity from the analysis. To allow for comparison of the impact of different factors, we standardized the variables hour of day (centered around 1200 h), temperature, light intensity, and nautical dawn (www.timeanddate.de) by centering with respect to their root mean square. Values for the abiotic factors were incorporated on an hourly basis. We only included data for days 2-5 of each experiment as too few flies eclosed on the other days of the experiments. The R library “GLMMadaptive” version 0.6-5 (Rizopoulos, 2019) was used for model fitting with the fixed explanatory variables genotype (factorial), hour and hour², temperature, light intensity, and nautical dawn nested into the random factor “experimental group” and with the dependent variable the proportion of flies emerging within a given daily hour out of those flies that had not yet emerged before on this day. We ignored interactions of higher than second order; the interaction term between hour and hour² was not included in the model. We selected the best statistical model by backward simplification (Crawley, 2013), starting with the most complex model and removing effects of weak significance until ANOVA model comparison yielded a significant difference, indicating that further simplification would result in a substantial worsening of the model.

## Results

### Eclosion Rhythmicity Under Laboratory Light and Temperature Entrainment in WT_CS_ and *per*^*01*^ Clock Mutants

**Figure 1.**
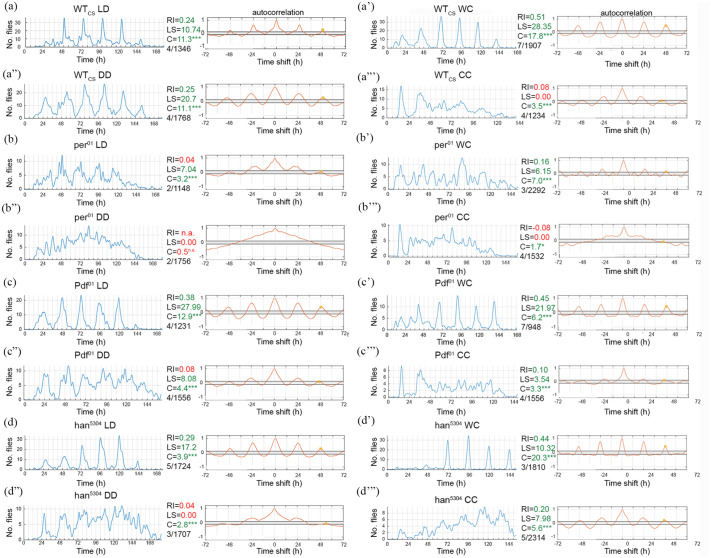
Eclosion profiles and autocorrelation analysis of WT_CS_, *per01*, and PDF signaling mutants under laboratory conditions. Left half: light entrainment (LD12:12), right half: temperature entrainment (WC12:12). WT_CS_ flies eclose rhythmically under ZT conditions (a-a′) as well as under DD (a″), yet quickly loose rhythmicity under constant temperature (a‴). In contrast, *per01* clock mutants show impaired rhythmicity under all conditions (b-b′: ZT conditions, b″-b‴: constant conditions). Under WC entrainment, however, ultradian rhythmicity appeared with a period of 12 h (b‴). Flies lacking either PDF (c-c‴) or the PDF receptor (*han5304*, d-d‴) eclose rhythmically during ZT conditions, but increasingly lose rhythmicity during constant conditions (c″-c‴, d’’-d‴). (***= < 0.001), #/# indicates *N* experiments/*n* flies. Abbreviations: WT_CS_ = wild-type Canton S; LD = light:dark; DD = constant darkness; WC = warm:cold; RI = rhythmicity index; LS: Lomb-Scargle; C = cosinor amplitude and zero-amplitude test significance level.

We first characterized the temporal eclosion pattern of wild-type Canton S (WT_CS_) and *per*^*01*^ clock-mutant flies under 12 h light:12 h dark (LD12:12) and 12 h warm:cold (WC12:12) entrainment. In LD, WT_CS_ flies showed typical daily eclosion profiles with bouts of eclosion events during a gate around ZT0 (lights-on), with strongly reduced or absent eclosion activity in the second half of the day (ZT12-23). WT_CS_ control flies were rhythmic under LD12:12 (Figure 1a) and maintained rhythmicity in DD after light entrainment (Figure 1a″). However, *per*^^*01*^^ mutants eclosed arrhythmically under DD—and at best weakly rhythmic, RI < 0.1 (arrhythmic), LS power = 7.04, cosinor amplitude = 3.2—in LD (Figure 1b and 1b″). These results are in line with previous studies (Konopka, 1972; Pittendrigh, 1954; Qiu and Hardin, 1996) which found a functional *per* gene to be required for eclosion rhythmicity under LD and DD conditions.

Under temperature entrainment, WT_CS_ eclosed rhythmically (Figure 1a′) with strong dampening in CC (Figure 1a‴) as reported previously (Konopka, 1972). The *per*^^*01*^^ mutants showed rhythmicity under WC conditions in line with an earlier report (Konopka, 1972), yet with a strong ultradian component with a period length of around 12 h (Figure 1b′). We speculate that this most likely represents a masking response to the changing temperature gradient and does not represent endogenously driven rhythmicity. In CC, *per*^^*01*^^ mutants turned arrhythmic (Figure 1b‴).

### Weaker Eclosion Rhythmicity Under Quasi-natural Conditions in Wild-type Flies

**Figure 2.**
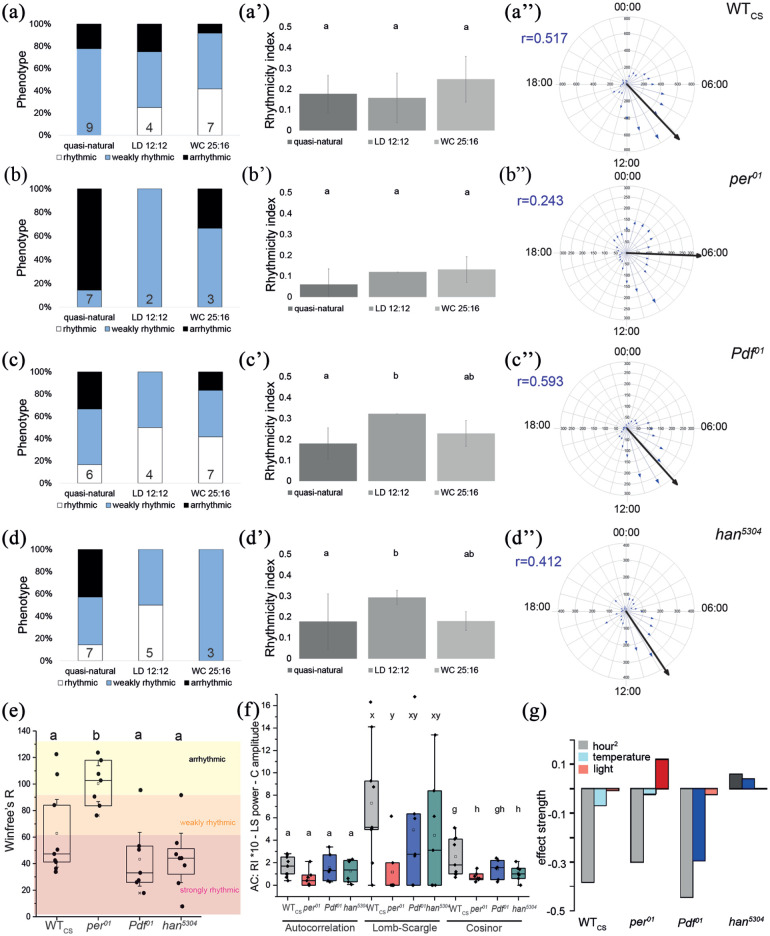
Autocorrelation analysis of eclosion rhythmicity under quasi-natural conditions in (a) WT_CS_, (b) *per01*, (c) *Pdf01*, and (d) *han5304* flies. The left column (a-d) summarizes the rhythmicity of the individual experiments under quasi-natural conditions (see Suppl. Table 1) and in LD12:12 and WC12:12 entrainment in the laboratory (see Figure 1). Numbers in the columns indicate *N*. The middle column (a′-d′) shows the mean RI ± SD for the different conditions. A Lomb-Scargle analysis of the data is shown in Supplementary Figure 1. The right column (a″-d″) shows circular plots of the data with mean vector (large black arrow) and sum of eclosed flies per hour over all experiments under quasi-natural conditions (small blue arrows). (e) Winfree’s RI for the different genotypes under quasi-natural conditions. (f) Comparison of the outdoor results obtained by autocorrelation (RI, left columns), Lomb-Scargle (power, middle columns), and cosinor (amplitude, right columns) analyses. While differences in RI between genotypes are not statistically significant, there is a significant difference in the Lomb-Scargle power and cosinor amplitude between *per01* and WT_CS_ flies (*p* < 0.05). (g) Effect size for parameters that show a significant interaction effect with the genotype. Independent variables scaled to mean; hour is scaled to 1200 h = 0. Darker colors indicate significant differences in WT_CS_ controls. Note that effect strengths cannot directly be compared between factors. Small letters indicate statistical significance (*p* < 0.05). a′ has already been published in a different context (Ruf et al., 2017). Abbreviations: WT_CS_ = wild-type Canton S; LD = light: dark; WC = warm: cold; RI = rhythmicity index.

To test for eclosion rhythmicity under natural conditions, we next assayed eclosion rhythmicity outdoors. Puparia were directly exposed to the ambient changes in temperature, humidity, and indirect changes in light intensity without interfering plastic or glass interfaces. Under these quasi-natural conditions, WT_CS_ flies eclosed rhythmically in the majority of experiments, 78% based on autocorrelation, 89% based on LS, and 67% based on cosinor (Figure 2a; Suppl. Fig. 1A, *n* = 9), as was expected from flies with intact circadian clock receiving continuous zeitgeber information. The proportion of rhythmic experiments was similar compared with flies raised and monitored under light-dark (LD12:12) conditions in the laboratory, 75% or 100%, respectively (*n* = 4, Figure 2a; Suppl. Fig. 1A). The mean daily hour for eclosion over all WT_CS_ experiments was 0906 h ± 0423 SD (*n* = 5730), with a mean vector length (*r*) of 0.517 (Figure 2a‴) as obtained by circular statistics. Thus, the eclosion of WT_CS_ flies does not concur with sunrise (mean: 0623 h) but occurs considerably later.

Under laboratory WC12:12 conditions, the proportion of rhythmic experiments for WT_CS_ was increased to 92% (autocorrelation, Figure 2a) and 100% (LS; Suppl. Fig. 1A), respectively. Compared with quasi-natural and LD conditions, the rhythmicity was slightly but not significantly increased under WC conditions (Figure 2a′), suggesting that stable temperature cycles promote robust cycling of eclosion rhythms. In line, the strongest rhythm under quasi-natural conditions occurred when the amplitude and shape of temperature oscillations remained stable over the course of the experiment (6 days) within a range of 15-30 °C (Suppl. Fig. 2a).

High temperatures (max. 35 °C) and mild nights above 20 °C had little effect on peak eclosion time (ψ_PK_), while a stronger correlation between minimum day temperature and ψ_PK_ (*r* = 0.383, *p* < 0.001) was found (Suppl. Fig. 3). The circular-linear correlation between maximum day temperature and peak eclosion time was only weak (ψ_PK_, *r* = 0.212, *p* = 0.056). In both laboratory (Pittendrigh, 1954; Zimmerman et al., 1968), and seminatural (Prabhakaran and Sheeba, 2013) experiments under tropical conditions, ψ_PK_ delayed with decreasing temperature, in line with our results. Yet, ψ_PK_ advanced with increasing temperature (Pittendrigh, 1954; Prabhakaran and Sheeba, 2013; Zimmerman et al., 1968), which was not observed in our experiments under temperate conditions.

### Eclosion Rhythmicity Is Impaired in *per*^*01*^ Flies Under Quasi-natural Conditions

Under quasi-natural temperate conditions, *per*^^*01*^^ flies eclosed arrhythmically in the majority of experiments (RI < 0.1: 86%, LS power and cosinor zero-amplitude test = 0: 71%, *n* = 7; Figure 2b; Suppl. Fig. 1B, Suppl. Table 1), although these flies received continuous zeitgeber information. As a consequence, the mean RI and LS power under quasi-natural conditions were markedly reduced compared to laboratory conditions (Figure 2b′, Suppl. Fig. 1B’). However, also under temperate conditions, rhythmic eclosion occurred in *per*^^*01*^^ flies, albeit in a minority of experiments (RI > 0.1: 14% of experiments, LS power and cosinor zero-amplitude test: 29% of experiments; Figure 2b; Suppl. Fig. 1B, Suppl. Table 1). Eclosion still appears to be gated in *per*^^*01*^^ flies under quasi-natural conditions: hardly any eclosion occurred in the afternoon, even though the “forbidden” phase was smaller and did not extend into the first half of the night as in WT_CS_ (Figure 2a″ and 2b″). Consequently, the eclosion gate was considerably wider when compared with WT_CS_ flies, and eclosion events occurred more frequently especially during the second half of the night (Figure 2b″). The mean eclosion time was around 3 h earlier than in WT_CS_ flies with high variance, 0608 h ± 0625 SD (*n* = 2449), *r* = 0.243 (Figure 2b″). This time is considerably close to the mean time of sunrise (0623 h), suggesting that eclosion in *per^*01*^* flies bears a stronger correlation to lights-on than in WT_CS_ controls. Earlier eclosion is not unexpected in *per*^^*01*^^ flies as circadian gating leads to a delay in eclosion or development of those flies that become ready to execute the final step(s) during the time the gate is closed.

To formally test for eclosion gate width, we calculated Winfree’s *R* (Figure 2e (Winfree, 1970)), another measure used for eclosion rhythmicity. The median *R* of *per^01^* mutant flies of 102.3 ± 6.7 SD (*n* = 7) was significantly higher (*p* = 0.04) than in WT_CS_ (47.3 ± 11.1 SD, *n* = 9) under quasi-natural conditions, supporting a more uniform eclosion and a significantly wider gate in the clock mutant. Winfree’s *R* for the laboratory data indicated arrhythmicity under LD12:12 (*R* = 117.1 ± 4.8 SD, *n* = 2) and weak rhythmicity (*R* = 84.1 ± 4.5 SD, *n* = 3) under WC12:12 conditions for *per^*01*^* flies. Combined, the *R* values suggest that the presence of abiotic zeitgeber under temperate quasi-natural conditions does not mitigate the impaired gate width and rhythmicity of *per*^^*01*^^ flies. This finding is in contrast to observations under tropical seminatural conditions where an eclosion gate of *per*^^*01*^^ flies became visible under seminatural conditions but was absent under LD conditions in the laboratory (De et al., 2012). Also for WT_CS_ controls, the presence of the full complement of abiotic factors did not improve the *R* value compared with either LD (*R* = 47.8 ± 8.5 SD, *n* = 4) or WC conditions (*R* = 25.0 ± 3.9 SD, *n* = 7).

We note that the rhythmic quasi-natural experiments in *per*^^*01*^^ mutants occurred under stable temperature amplitudes with temperate highs (Suppl. Fig. 2A′). However, in most experiments, including others with zeitgeber changes of equal stability, eclosion was arrhythmic in *per*^^*01*^^ mutant flies (Suppl. Fig. 2B′, Suppl. Table 1), while WT_Cs_ controls maintained rhythmicity (Suppl. Fig. 1B, Suppl. Table 1). Compared with WT_CS_ flies, *per*^^*01*^^ mutants showed a stronger circular-linear correlation between maximum day temperature and ψ_PK_ (*r* = 0.335, *p* = 0.003) and a weaker correlation between minimum day temperature and ψ_PK_ (*r* = 0.262, *p* = 0.026). This seems to parallel the increased responsiveness to higher temperatures of *per^*01*^* flies in locomotor activity (Menegazzi et al., 2012).

### Impaired Eclosion Timing Under Quasi-natural Conditions in *per*^*01*^; *tim*^*01*^ Mutant Flies

The results above showed that *per^*01*^* mutants eclosed mostly arrhythmically under quasi-natural temperate conditions. To verify this effect of an impaired molecular clock, we conducted outdoor eclosion experiments in a subsequent year, using *per^*01*^*; *tim*^^*01*^^ double mutants. Like for *per* (Blanchardon et al., 2001; Konopka and Benzer, 1971), mutation in the *timeless* (*tim*) gene (Sehgal et al., 1994) as well as overexpression of *tim* in the prothoracic gland disrupts eclosion rhythmicity (Myers et al., 2003; Selcho et al., 2017). Under LD laboratory conditions, *per^*01*^*; *tim*^^*01*^^ flies showed higher rhythmicity than *per*^^*01*^^ flies (RI = 0.33, LS power = 17.9, cosinor amplitude = 2.78, zero-amplitude test *p* < 0.00001; Suppl. Fig. 4A), but similar to *per*^^*01*^^ turned arrhythmic under DD (RI = 0.20, yet second peak in autocorrelation lost, LS power = 0, cosinor zero-amplitude test *p* = 0.39; Suppl. Fig. 4A′). During the outdoor experiments, the frequent very high temperatures during 2016 unfortunately lead to a high developmental mortality. Out of the four successful experiments (mean RI = 0.14 ± 0.19 SD, mean LS power = 2.57 ± 2.81 SD, mean cosinor amplitude = 0.98 ± 0.63), one was arrhythmic (RI = −0.12, LS power = 0, cosinor zero-amplitude test, *p* > 0.05), two were weakly rhythmic (R = 0.12 and 0.13, LS power = 0.15 and 3.23, cosinor amplitude <2), and one was strongly rhythmic (RI = 0.43, LS power = 6.8, cosinor amplitude = 1.6) with comparably stable zeitgeber amplitudes during these experiments (Suppl. Table 1). In contrast to *per^*01*^* flies, *per*^^*01*^^; *tim*^^*01*^^ mutants showed a wild-type-like mean eclosion time of 0934 h ± 0534 SD (*n* = 1918), with a vector length (*r*) of 0.345 (Suppl. Fig. 4C). Consistent with the results from *per*^^*01*^^ flies, the eclosion gate was considerably wider compared with WT_CS_ flies, Winfree’s *R* = 87.7 ± 9.1, a value close to arrhythmicity (*R* > 90). The wider eclosion gate and Winfree’s *R* are statistically similar to *per*^^*01*^^ mutant flies (Suppl. Fig. 4D), with *per*^^*01*^^; *tim*^^*01*^^ flies also showing extended eclosion activity not only into the second half of the night but also into early afternoon.

### Impaired PDF Signaling Has Little Effect on Eclosion Rhythmicity Under Quasi-natural Conditions

Next, we monitored eclosion rhythmicity under laboratory and quasi-natural conditions in *Pdf*^^*01*^^ *and Pdfr (han*^*5304*^) mutant flies which are defective in PDF signaling. PDF is a neuropeptide signal released by a subset of circadian pacemaker cells in the brain (the PDF^+^ small and large lateral ventral neurons [s- and lLN_v_s]) which is received by the PDF receptor (PDFR) encoded by *han* and expressed by a large set of central clock neurons (Helfrich-Förster, 1997; Hyun et al., 2005; Im and Taghert, 2010; Shafer et al., 2008). PDF signaling is important to maintain stable phase and Ca^2+^ activity relationship between central clock neurons (Liang et al., 2017). Besides this clock-internal function, PDF is also a major output factor of the circadian clock (Krupp et al., 2013; Nagy et al., 2019; Shafer and Yao, 2014). If the molecular clockwork is required for eclosion rhythmicity under natural conditions, we expected PDF signaling mutants to show rather normal eclosion rhythmicity under quasi-natural conditions, as the individual groups of pacemaker neurons are kept in stable phase relationship by the zeitgebers. If PDF is also required as an output factor, then we expected impaired eclosion rhythmicity.

Consistent with the literature (Blanchardon et al., 2001; Myers et al., 2003; Selcho et al., 2017), PDF signaling mutants were rhythmic under laboratory LD12:12 conditions (Figure 1c and 1d), but became arrhythmic after 2-3 days in DD (Figure 1c″ and 1d″) presumably since the individual groups of pacemaker cells lose their proper sequence of activity (Selcho et al., 2017). When entrained in LD and transferred to DD during an early pupal stage at 20 °C, both *Pdf^01^* and *han*^*5304*^ flies lost eclosion rhythmicity already during the first 2 days of monitoring (data not shown). *Pdf^01^* and *han^5304^* mutants also eclosed rhythmically under WC conditions (Figure 1c′ and 1d′), and maintained residual rhythmicity under CC (Figure 1c‴ and 1d‴), albeit with a strongly altered eclosion pattern lacking the clear drop in eclosion events during the second half of the day.

Under quasi-natural conditions, *Pdf^*01*^* and *Pdfr* mutants (*han*^*5304*^) were predominantly rhythmic (67% and 57%; RI > 0.1, LS power > 0; 67% and 85% cosinor zero-amplitude test; *n* = 6, 7), although more arrhythmic experiments were observed than in WT_CS_ controls. The mean eclosion time over all experiments was very similar to WT_CS_, with 0913 h ± 0354 SD and a vector length (*r*) of 0.593 for *Pdf*^^*01*^^ flies (*n* = 1918) and 0944 h ± 0505 SD and a vector length (*r*) of 0.412 for *han*^*5304*^ flies (*n* = 1918). Also, Winfree’s *R* showed strong rhythmicity in 85% of the experiments for *Pdf*^^*01*^^ (*R* = 43.3 ± 26.4 SD, *n* = 7) and *han*^*5304*^ (*R* = 44.3 ± 24.2 SD, *n* = 8) flies. For both *Pdf*^^*01*^^ and *han*^*5304*^ flies, mean *R* was significantly smaller (*p* = 0.002) than for *per^*01*^* mutants (100.3 ± 17.6 SD; Figure 2e). A correlation between ψ_PK_ peak eclosion time and maximum/minimum day temperature was found for *han*^*5304*^ PDF receptor mutants (maximum: *r* = 0.293, *p* = 0.008; minimum: *r* = 0.601, *p* < 0.001), but was missing in *Pdf*^^*01*^^ flies.

Taken together, these data indicate that natural zeitgeber under temperate conditions can generally compensate for a loss of PDF signaling (*Pdf*^^*01*^^, *han*^*5304*^) but not for the loss of a functional clock (*per*^^*01*^^, *per*^^*01*^^; *tim*^^*01*^^) in eclosion timing. Obviously, a functional endogenous clock is required for robust rhythmic and properly gated eclosion behavior under variable quasi-natural conditions.

### Combined Data Over the Season Support the Conclusion From Individual Experiments

**Figure 3.**
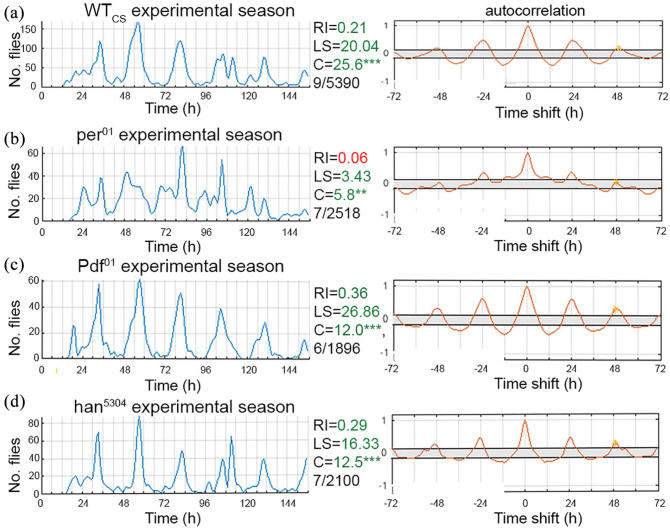
Combined eclosion profiles (left) and autocorrelation (right) under quasi-natural conditions. All experiments during the experimental season are combined, ignoring differences in day length and time of sunrise and sunset between the individual weeks. (a) WT_CS_, (b) *per01*, (c) *Pdf01*, and (d) *han^5304^* flies. Note the broader eclosion windows and less-defined eclosion peaks as well as arrhythmic RI and low LS power in the *per01* mutants. (*** = <0.001), #/# indicates *N* experiments/*n* flies. Abbreviations: WT_CS_ = wild-type Canton S; RI = rhythmicity index; LS: Lomb-Scargle; C = cosinor amplitude and zero-amplitude test significance level.

We next combined all data from the various experiments over the season. Confirming the results from the individual experiments, this revealed surprisingly clear rhythmicity (RI = 0.21-0.36, LS power = 16.33-26.86, cosinor amplitude = 12.0-24.6, zero-amplitude test *p* < 0.00001), distinct ψ_PK_, and well-defined gates in WT_CS_, *Pdf^*01*^*, and *han5304* flies (Figure 3a-3d). These results suggest that eclosion under natural conditions is largely determined by the clock and not by sunrise or the correlated rise in temperature, as sunrise varied quite considerably from 0529 to 0720 h, with a photoperiod ranging from LD16:8 to 11:13 during the experimental season. In contrast, the combined eclosion profile of *per*^^*01*^^ mutants was classified as arrhythmic (RI = 0.06) or weakly rhythmic (LS power = 3.43, cosinor amplitude = 5.8, zero-amplitude test *p* = 0.0015), with broader gates and less-defined eclosion peaks (Figure 3b). Nevertheless, though ψ_PK_ in *per^*01*^* mutants represents a considerably smaller proportion of flies than in the other genotypes, it still coincided well with ψ_PK_ of WT_CS_ and the PDF signaling mutants (Figure 3). Though recorded in a different year and thus with restricted comparability, the combined profile of *per*^^*01*^^; *tim*^^*01*^^ flies looks similar to *per*^^*01*^^ flies with similar rhythmicity scores (RI = 0.06, LS power = 16.77, cosinor amplitude = 3.8, zero-amplitude test *p* < 0.00001; Suppl. Fig. 4B).

### Humidity as a Zeitgeber Is Unable to Entrain Eclosion Rhythmicity

**Figure 4.**
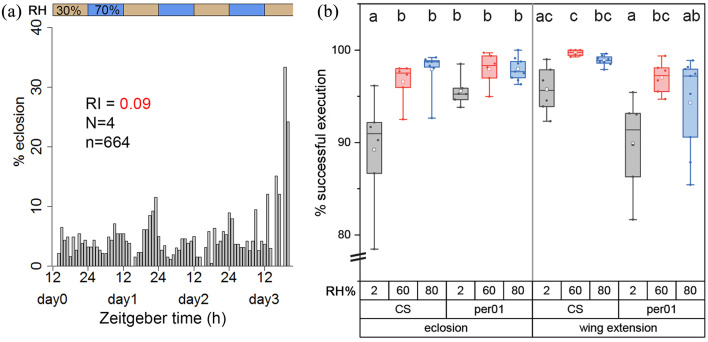
WT_CS_ flies were kept in DD at a humidity cycle of 12 h 30% and 12 h 70% RH. (a) Despite the humidity cycle, flies eclosed arrhythmically, indicating that humidity changes are unable to entrain eclosion rhythmicity. (b) Percentage of flies that either successfully eclosed (left) or extended wings among those that did eclose (right). Small letters indicate statistical significance (*p* < 0.05). Abbreviations: DD = constant darkness; RH = relative humidity; CS = Canton S.

Temperature and RH are inversely correlated, making it difficult to separate the influence of these parameters on eclosion timing under quasi-natural conditions. Humidity has been suggested to modulate eclosion timing of *Drosophila* (De et al., 2012), but there is no direct evidence for this assumption and it is unclear whether humidity acts as a zeitgeber for *Drosophila*. We therefore tried to entrain WT_CS_ flies to humidity cycles of 12 h 70% and 12 h 30% RH under constant red light (λ = 635 nm) at 20 °C throughout the entire development. Under these conditions, flies eclosed arrhythmically (mean RI = 0.09 ± 0.14, *N* = 4, *n* = 664) without distinguishable preference for either the humid or dry phase (Figure 4a). This finding shows that *Drosophila* is unable to entrain to humidity cycles and hence does not use humidity as a zeitgeber.

The timing of eclosion to the more humid morning hours is regarded as an adaptation to reduce water loss and allow for proper wing unfolding as long as the cuticle is untanned (Clayton and Paietta, 1972; Pittendrigh, 1993). We therefore tested whether RH has an effect on eclosion timing or whether low RH might even prevent eclosion. WT_CS_ and *per*^^*01*^^ mutant flies were allowed to eclose at normal (60%), high (80%), and extremely low (2%) RH at 25 °C, and failure to eclose or to expand the wings upon successful eclosion was noted. Even at 2% RH, ≥88% of flies eclosed successfully and expanded their wings (Figure 4b; WT_CS_: *N* = 6, *n* = 829; *per*^^*01*^^: *N* = 6, *n* = 2047). Only for WT_CS_ eclosion was this percentage significantly lower than in both controls at 60% and 80% RH (Figure 4b, *p* < 0.05; WT_CS_: 60% RH: *N* = 6, *n* = 1581; 80% RH: *N* = 8, *n* = 3532; *per*^^*01*^^: 60% RH: *N* = 6, *n* = 1936; 80% RH: *N* = 9, *n* = 3340). These results speak against a direct inhibition of eclosion by low RH within natural habitat conditions of *D. melanogaster* and show that low humidity does not significantly affect wing expansion in the fruit fly. This is against earlier assumptions (Pittendrigh, 1954), but in line with results from the much larger onion fly *Delia antiqua* (Tanaka and Watari, 2009).

### Eclosion Timing of WT_CS_ but Not Clock-related Mutants Is Largely Unaffected by Day-to-Day Variation in the Amplitude and Absolute Level of Abiotic Zeitgebers

To assess the correlation between time of eclosion and abiotic zeitgebers, and to evaluate eclosion probabilities in response to changes in environmental variables, we applied a logistic regression model. We included time of day (=hour and hour^2^), temperature, light intensity, and nautical dawn as environmental variables and genotype as a categorial predictor, and ignored interactions of higher than second order. We selected the best statistical model by backward simplification (Crawley, 2013), starting with the most complex model. Then, effects of weak significance were removed in a stepwise manner until ANOVA model comparison yielded a significant difference which indicates that further simplification would result in a substantially inferior model. Examples of the actual data for each day and genotype plotted against the best model are shown in Supplementary Figs. 5 and 6 which illustrate the relative contribution of the different factors.

All environmental variables contributed significantly (in particular via interaction effects) to the explanation of the observed temporal patterns. In general, eclosion timing is dominated by hour of the day (Figure 2g; Suppl. Table 2), with an overall tendency of increasing eclosion probabilities over the course of the day but a more or less pronounced peak for eclosion probability before noon (Suppl. Figs. 5 and 6). This does not exclude that the majority of animals eclose already at an earlier time of the day as the probability that an individual does not eclose before hour *h* = *T* is $\prod_{h=1}^{T-1}\left(1-ph\right)$. Time of nautical dawn had a moderate effect with earlier eclosion when sun rises earlier. While temperature and light intensity acted as weaker main factors, both were involved in strong interaction effects with hour of the day (Suppl. Table 2).

The quadratic time component (hour^2^) had a high negative effect strength in WT_CS_, as well as in *per*^^*01*^^ and *Pdf*^^*01*^^ mutant flies (Figure 2g; Suppl. Table 2). These negative values indicate a particular peak eclosion phase every day, which seems to be hardly present in *han*^*5304*^ flies (Figure 2g). Light, in contrast, had little effect on eclosion except for *per*^^*01*^^ mutants (Figure 2g), while temperature had a significant and opposing effect on *Pdf*^*01*^ and *han*^*5304*^ flies (Figure 2g). These results support the experimental finding that eclosion timing in WT_CS_ flies is strongly driven by the endogenous clocks, while clock and PDF signaling mutants are more susceptible to follow changes of the main zeitgeber light and temperature, respectively. Importantly, possession of a functioning molecular clock (WT_CS_, *Pdf*^^*01*^^, *han*^*5304*^) seems to uncouple eclosion behavior from momentary changes in light intensity. The high effect strength of light on *per^*01*^* mutants (Figure 2g) might at least partially explain the significantly earlier eclosion time of *per^*01*^* mutants (Figure 2b″) with a mean around 0600 h, which is in between the mean time of nautical dawn (0504 h) and official sunrise (0623 h) during the experimental period. The stronger effect of temperature on *Pdf*^^*01*^^ mutants is remarkably similar to the stronger temperature dependency of the onset of evening locomotor activity in *Pdf*^*01*^ mutants under LD conditions (Miyasako et al., 2007). For locomotor activity, the PDF-expressing sLNvs are a central part of a light-entrainable oscillator that dampens the temperature effect on the phase of the clock neuron subsets constituting the temperature-entrainable oscillator (Miyasako et al., 2007). Also for eclosion timing, lack of PDF signaling may lead to a stronger contribution of the temperature-entrainable oscillator. This implies also a significant effect of temperature on *Pdfr* mutants (*han*^*5304*^), which indeed seems to be the case (Figure 2g; Suppl. Table 1).

## Discussion

Eclosion rhythmicity is a consequence of a developmental step gated in time. It occurs on the population level and can be characterized by its strength, period, phasing (ψ_PK_), and gate width (the time window during a day in which eclosion occurs). Our results provide evidence for a requirement of a functional molecular clock to maintain consistent and normal eclosion rhythmicity and gating under quasi-natural temperate conditions in the fruit fly *Drosophila*. Compared with wild-type flies, *per*^*01*^ clock mutants showed a significantly reduced rhythmicity strength, a significantly enlarged gate width, and a significant earlier mean eclosion time, while the period and phasing of ψ_PK_ were basically unaltered.

This contrasts with earlier results for *Drosophila* locomotor activity under seminatural conditions (Vanin et al., 2012). While *per^*01*^* mutants in the majority of our experiments eclosed mostly arrhythmically and significantly earlier than WT_CS_ controls, Vanin and colleagues report 95% rhythmic locomotor activity in *per*^^*01*^^ and *per^*01*^*; *tim*^^*01*^^ flies under quasi-natural temperate conditions, with an onset of morning activity indistinguishable between clock mutants and WT_CS_ controls (Vanin et al., 2012). Thus, circadian dominance seems to be markedly stronger for eclosion behavior than for locomotor activity. This suggests that precise timing of eclosion, a very crucial and behaviorally stereotyped one-time event during life history, might be more related to circadian fitness than the precise timing of recurrent and plastic locomotor activity.

At the same time, we note that in some of the individual experiments in *per*^^*01*^^ and *per*^*01*^; *tim*^*01*^ flies, eclosion was rhythmic as classified by various common analysis methods. Moreover, none of the eclosion profiles of *per*^*01*^ or *per*^*01*^; *tim*^*01*^ flies under quasi-natural conditions took the shape of Gaussian distribution. A Gaussian distribution of eclosion events is indicative of nongated developmental timing and is found in clock-mutant flies under constant conditions in the laboratory (Konopka, 1972; Konopka and Benzer, 1971; Qiu and Hardin, 1996; Sehgal et al., 1994). This suggests that natural zeitgebers to a small and even larger extent under certain uncharacterized conditions are able to compensate the loss of a functional clock for eclosion rhythmicity strength. In support, the modeling results suggest that light for *per*^*01*^ flies and temperature for PDF signaling mutants have a significantly higher effect on eclosion than in WT_CS_ controls.

Importantly still, gate width and mean time of eclosion are consistently and markedly altered in *per*^^*01*^^ mutants, and in the majority of experiments, *per*^^*01*^^ flies eclosed arrhythmically as classified by different means, showing that circadian dominance is prevailing under temperate conditions. Also, *per*^*01*^; *tim*^^*01*^^ showed an enlarged gate width. From these results, we conclude that circadian dominance cannot generally be regarded as vestigial for natural behavioral activity in day-active animals (Hazlerigg and Tyler, 2019), at least not for feeding/hunger-independent behaviors like eclosion. We speculate that the earlier and less precise timing of *per^*01*^* flies may lead to a misalignment with respect to the optimum time-of-day for eclosion, which should cause fitness costs. This idea needs to be tested in future studies and is based on findings in mice kept under natural conditions. In these mice, the mutant allele *Per2*^*BRDM*^ did not result in overall reduced fitness (Daan et al., 2011), yet a strong natural selection occurred against the *tau* allele of *Caseine kinase 1ε* (*Ck1ε*^*tau*^) (Spoelstra et al., 2016). *Ck1ε*^*tau*^ does not disrupt but accelerates the molecular clock, leading to a circadian misalignment to environmental cycles.

*D. melanogaster* originated in tropical Africa south of the Sahara, colonized Europe and Asia about 15,000 years ago, and was brought to the Americas and Australia some hundred (tropical America) to <200 years ago in conjunction with slave trade (David and Capy, 1988; Lachaise et al., 1988). As cosmopolitan species, *D. melanogaster* adapted to many different climates, from tropical to temperate regions. This adaption lead to latitudinal clines of different morphological and behavioral traits (David and Capy, 1988; Markow, 2015). Latitudinal clines were also found for *Drosophila* clock genes (see Hut et al., 2013; Kyriacou et al., 2008) and timing of the eclosion phase (Lankinen, 1986, 1993; Pittendrigh et al., 1991; Pittendrigh and Takamura, 1989).

It is therefore interesting to note that *per*^*01*^ mutant flies showed higher eclosion rhythmicity under seminatural tropical conditions in Bangalore (India) than under LD12:12 in the laboratory, based on the presence of an eclosion gate under seminatural, but not laboratory conditions (De et al., 2012). This is not contradicting our findings, as the prevailing weather conditions are much more stable in the tropics, with daily temperature curves more predictable and regular and having much reduced variability in day-to-day light intensity and temperature amplitudes compared with temperate conditions in Germany. During the course of our experiments, stable temperature amplitudes were rarely met. Moreover, in both studies, the eclosion profile was less robust than in controls, and eclosion gate width increased and the eclosion phase to a similar extend shifted forward into the end of night in *per*^*01*^ flies. Thus, the difference in eclosion rhythmicity and robustness between temperate and tropical conditions appear to be more in terms of magnitude and to a certain extent may reflect differences in the amplitude of changes in temperature or other abiotic factors. While a functional clock seems to be required under both tropical and temperate conditions for proper eclosion timing, the circadian dominance of eclosion obviously has gained in importance with the spread of *D. melanogaster* into temperate regions, ensuring rhythmic eclosion at the right time of the day under fluctuating weather conditions.

## Supplemental Material

sj-pdf-1-jbr-10.1177_0748730421998112 – Supplemental material for Natural Zeitgebers Under Temperate Conditions Cannot Compensate for the Loss of a Functional Circadian Clock in Timing of a Vital Behavior in Drosophila Click here for additional data file. Supplemental material, sj-pdf-1-jbr-10.1177_0748730421998112 for Natural Zeitgebers Under Temperate Conditions Cannot Compensate for the Loss of a Functional Circadian Clock in Timing of a Vital Behavior in Drosophila by Franziska Ruf, Oliver Mitesser, Simon Tii Mungwa, Melanie Horn, Dirk Rieger, Thomas Hovestadt and Christian Wegener in Journal of Biological Rhythms
