# Supplementary material for: Natural Zeitgebers Under Temperate Conditions Cannot Compensate for the Loss of a Functional Circadian Clock in Timing of a Vital Behavior in Drosophila
Source: J Biol Rhythms. 2021 Mar 22;36(3):271–85. doi: 10.1177/0748730421998112 (PMC8114442; doi:10.1177/0748730421998112)
Supplement: sj-pdf-1-jbr-10.1177_0748730421998112 – Supplemental material for Natural Zeitgebers Under Temperate Conditions Cannot Compensate for the Loss of a Functional Circadian Clock in Timing of a Vital Behavior in Drosophila [file sj-pdf-1-jbr-10.1177_0748730421998112.pdf]

Supplementary material

Supplementary Table 1. Individual autocorrelation rhythmicity indeces (RI), Lomb-Scargle power (LS) and Cosinor amplitude and significance level (C), as well as the number of flies per experiment under quasi-natural conditions. Total n=13,257 flies. * = p<0.05; ** = p<0.01; *** = p<0.001

	17.07.-24.07.2014				24.07.-31.07.2014				31.07.-07.08.2014				07.08.-14.08.2014				21.08.-28.08.2014			
Genotype	RI	LS	C	n	RI	LS	C	n	RI	LS	C	n	RI	LS	C	n	RI	LS	C	n
WT_{CS}	.04	0	$1.0^{n.s.}$	488	.21	4.95	1.8^{***}	292	.28	9.27	3.6^{***}	537	.1	8.74	4.2^{***}	736	.25	14.1	1.2^{*}	366
per^{01}									0	0	$0.6^{n.s.}$	576	0	0	$0.3^{n.s.}$	210	.21	1.99	1.0^{**}	222
Pdf^{01}	.04	0	$0.5^{n.s.}$	178					.14	2.06	2.4^{***}	323	.34	5.93	1.5^{***}	234	.12	0	$0.3^{n.s.}$	132
han^{5304}	.06	0	$0.0^{n.s.}$	280	.21	0	1.0^{*}	171	.23	6.12	2.1^{***}	241	.03	0	1.4^{**}	256	.22	8.39	0.6^{*}	135

	28.08.-04.09.2014				04.09.-11.09.2014				11.09.-18.09.2014				25.09.-02.10.2014			
Genotype	RI	LS	C	n	RI	LS	C	n	RI	LS	C	n	RI	LS	C	n
WT_{CS}	.17	1.97	$1.0^{n.s.}$	664	.07	5.15	5.1^{***}	1036	.27	16.31	4.1^{***}	764	.1	5.04	$0.7^{n.s.}$	582
per^{01}	.07	0	$0.6^{n.s.}$	258	.04	0	$0.5^{n.s.}$	298	.09	6.13	1.5^{***}	358	.01	0	$0.8^{n.s.}$	316
Pdf^{01}					.03	6.34	1.6^{***}	299	.2	16.79	2.2^{***}	354				
han^{5304}					.06	3.11	1.5^{**}	330					.35	10.39	0.9^{*}	291

	14.07.-21.07.2016				28.07.-03.08.2016				01.09.-07.09.2016				15.09.-22.09.2016			
Genotype	RI	LS	C	n	RI	LS	C	n	RI	LS	C	n	RI	LS	C	n
per^{01};*tim*^{01}	-0.12	0	0.5^{***}	105	.12	0.15	1.6^{***}	389	.43	6.8	1.6^{***}	332	.13	3.23	$0.2^{n.s.}$	123

Supplementary Table 2. Summary of parameter estimates for the best fitting model. First column specifies estimated model parameters for the reference genotye CS±s.e.m. (significant values plotted in bold), the other values in the genotype colums significant differences in parameter estimates for the other genoytpes. The last column lists significant interaction terms between the environmental covariates.

WT_{CS}		*per*^{01}	*Pdf*^{01}	*han*^{5304}	Interactions between environmental variables
**-1.29±.1**	intercept	--	--	-0.56	
**0.55±.03**	hr	--	--	--	temperature (-.31***), light (-.53***), dawn (-.28***)
**-0.38±.07**	hr^2	--	--	0.44	temperature (.15***), light (-.18**)
-0.07±.05	temperature	--	-0.23	0.11	light (.07**)
0.01±.04	light	0.12	--	--	dawn (-.13***)
**-0.14±.06**	dawn	--	--	--	

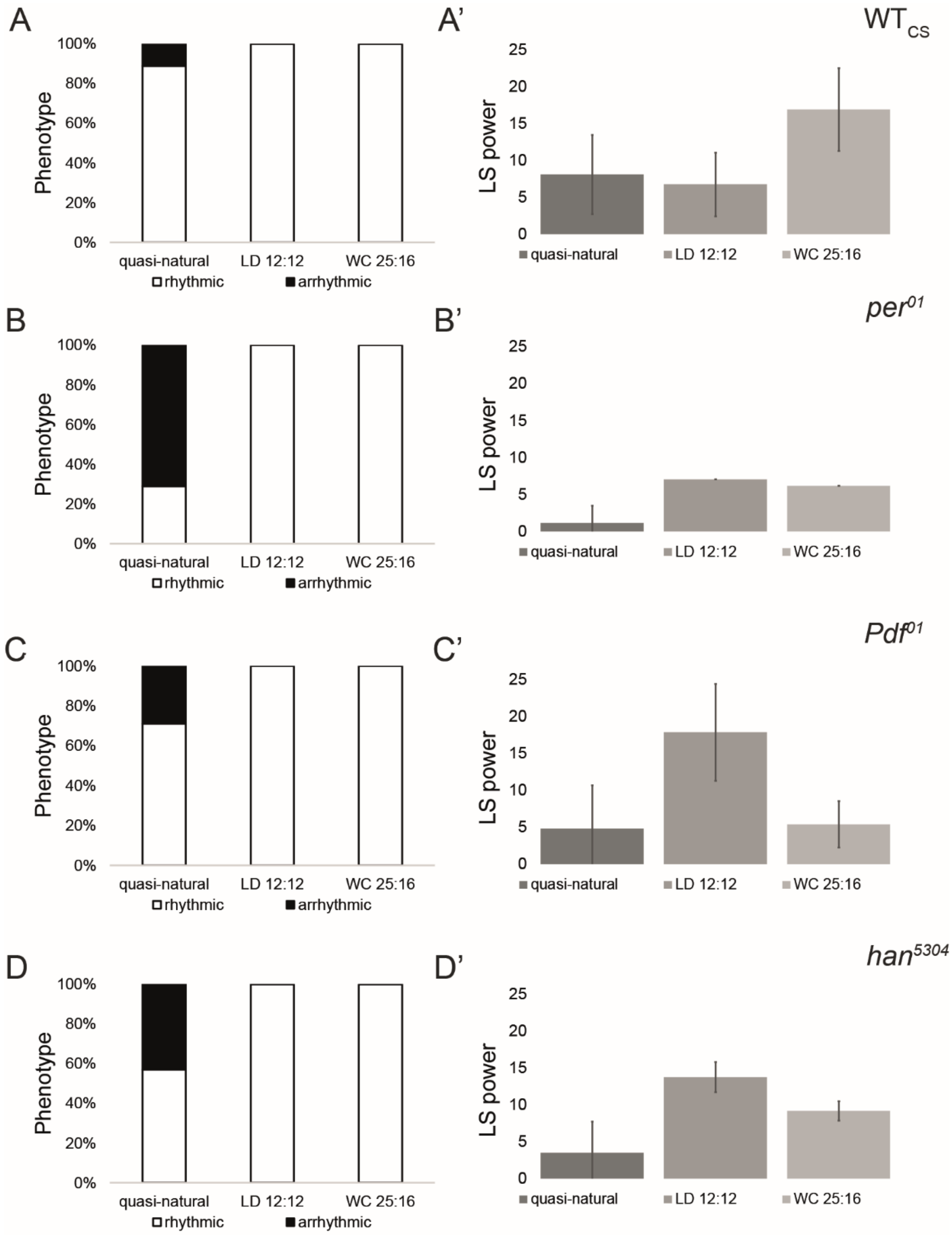

Supplementary Fig 1: Lomb-Scargle analysis of eclosion rhythmicity under quasi-natural conditions in **A)** WT$_{CS}$, **B)** *per^{01}*, **C)** *Pdf^{01}* and **D)** *han^{5304}* flies. The left column **(A-D)** summarises the rhythmicity of the individual experiments under quasi-natural conditions (see Suppl Table 1), and in LD12:12 and WC12:12 entrainment in the laboratory (see Fig 1). The right column **(A'-D')** shows the mean Lomb-Scargle power ± s.d. for the different conditions.

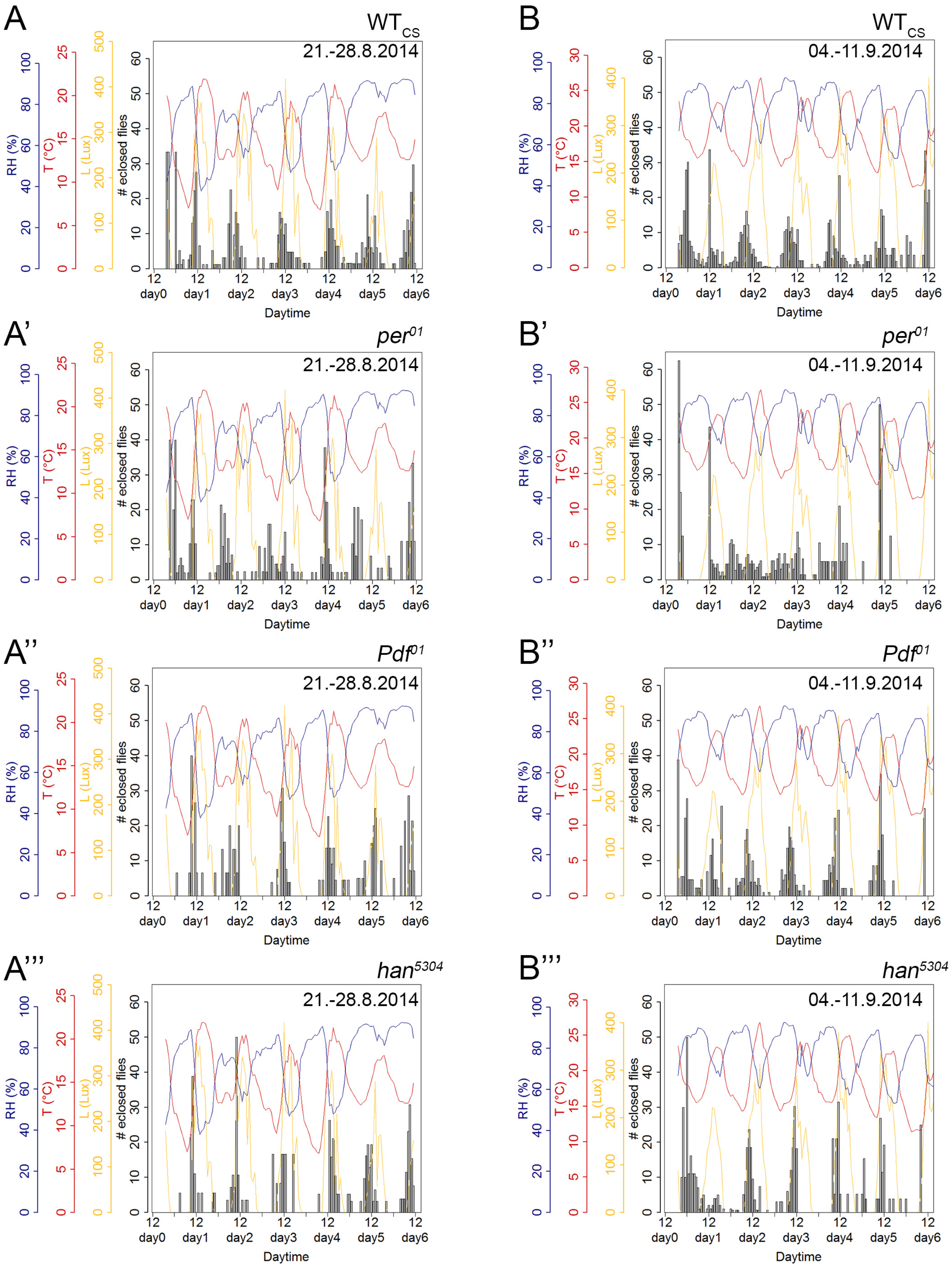

Supplementary Fig 2: Original eclosion profiles of WT_{CS} (top row, A-B) and *per*^{01} mutant flies (bottom row, A'-B') for two different weeks end of August (A-A') and beginning of September (B-B'). Relative humidity (RH) is shown in blue, Temperature (T) in red, and light intensity (L) in yellow.

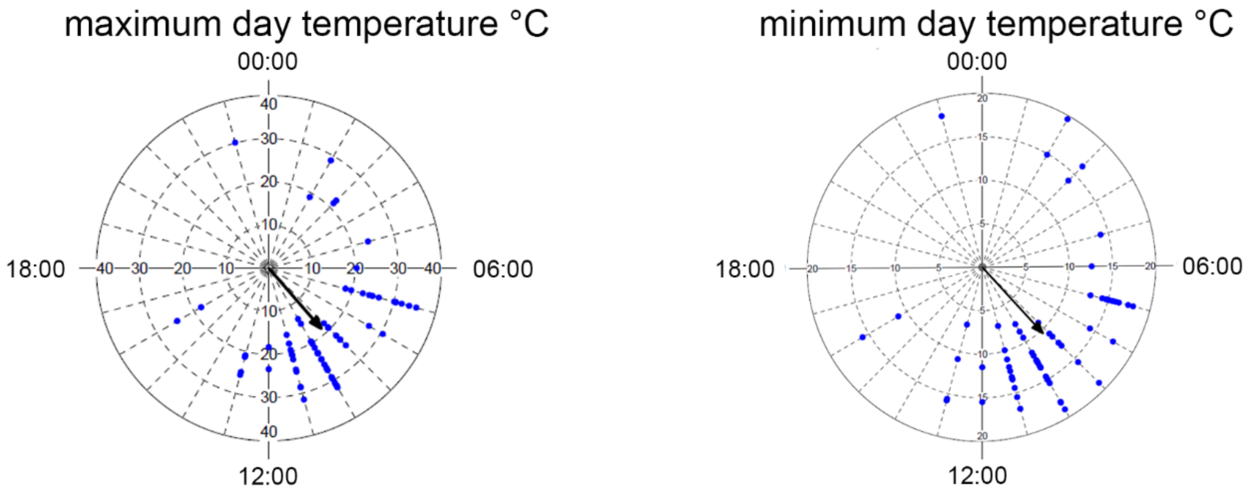

Supplementary Fig 3: Circular correlation of the time of eclosion (blue dots) with the daily maximum (left) and minimum temperature (right) of WT_{CS} flies with mean vector (black arrow) over all experiments under quasi-natural conditions.

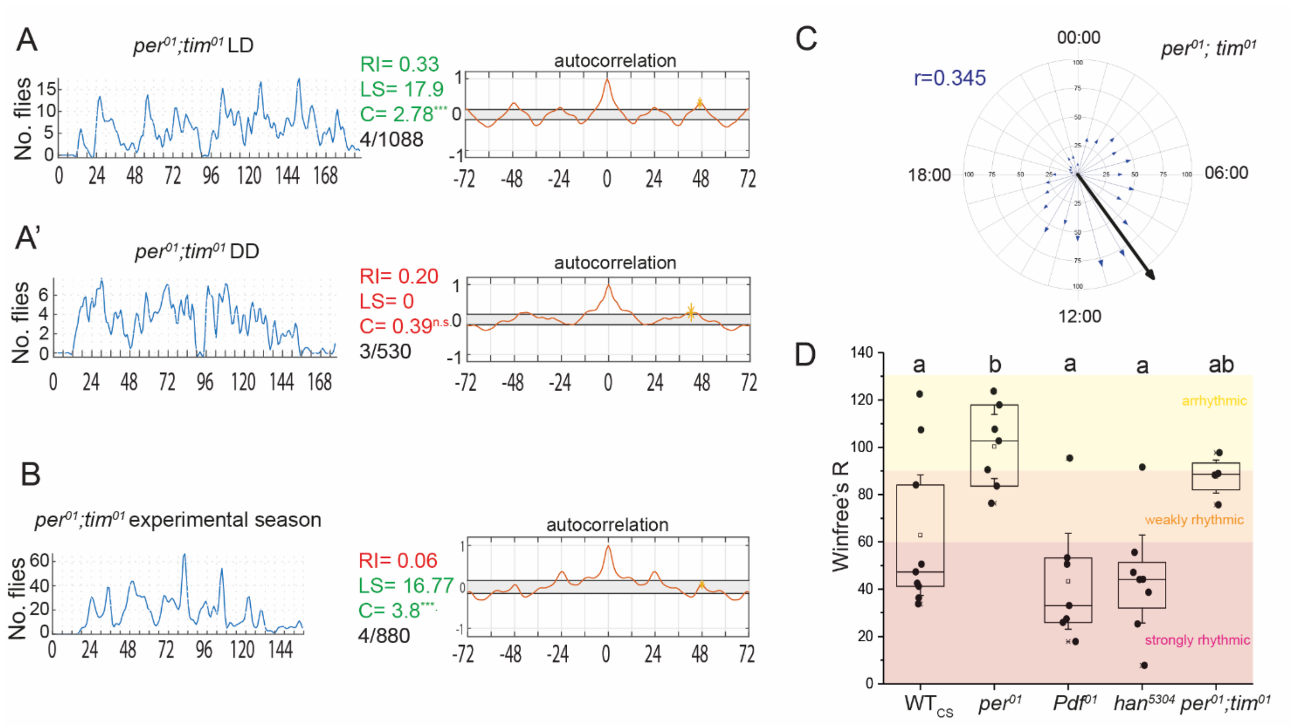

Supplementary Fig 4: A-B) Eclosion profiles (left) and autocorrelation analysis of *per*^{01}*; tim*^{01} flies (right). Flies were kept under laboratory conditions in LD (A) or DD (A'). B) Combined results for the experiments under quasi-natural conditions. All experiments during the experimental season are combined, ignoring differences in day length and time of sunrise and sunset between the individual weeks. RI= autocorrelation rhythmicity index; LS: Lomb-Scargle power; C= cosinor amplitude and zero-amplitute test significance level (***= <0.001), #/# indicates N experiments/n flies. C) Circular plots of the data with mean vector (black arrow) and sum of eclosed flies per hour over all experiments under quasi-natural conditions (blue arrows). E) Winfree's rhythmicity index for the different genotypes under quasi-natural conditions as in Fig 2E, but results of *per*^{01}*;tim*^{01} flies added. It is visible that the double mutants have a similarly enlarged eclosion gate as *per*^{01} mutants.

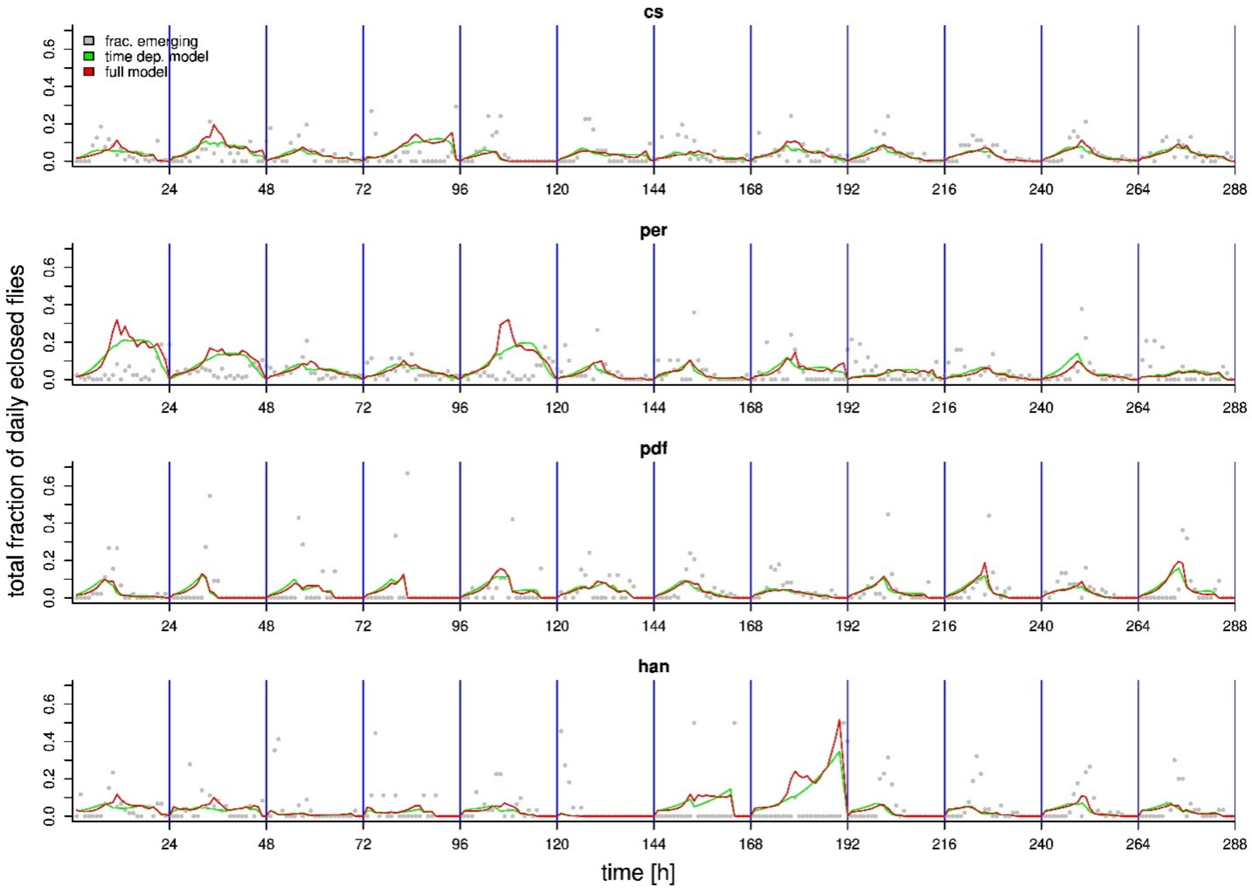

Supplementary Fig 5. Comparison of model predictions between four genotypes. Grey points show the fraction/hour of flies eclosing during a day. The green line shows the predicted emergence based on hour and hour^2 only whereas the red line shows the curve for the best fitting model also accounting for the effects of temperature, light and time of sun-rise (dawn). For clarity the figure shows only data and model for 12 days.

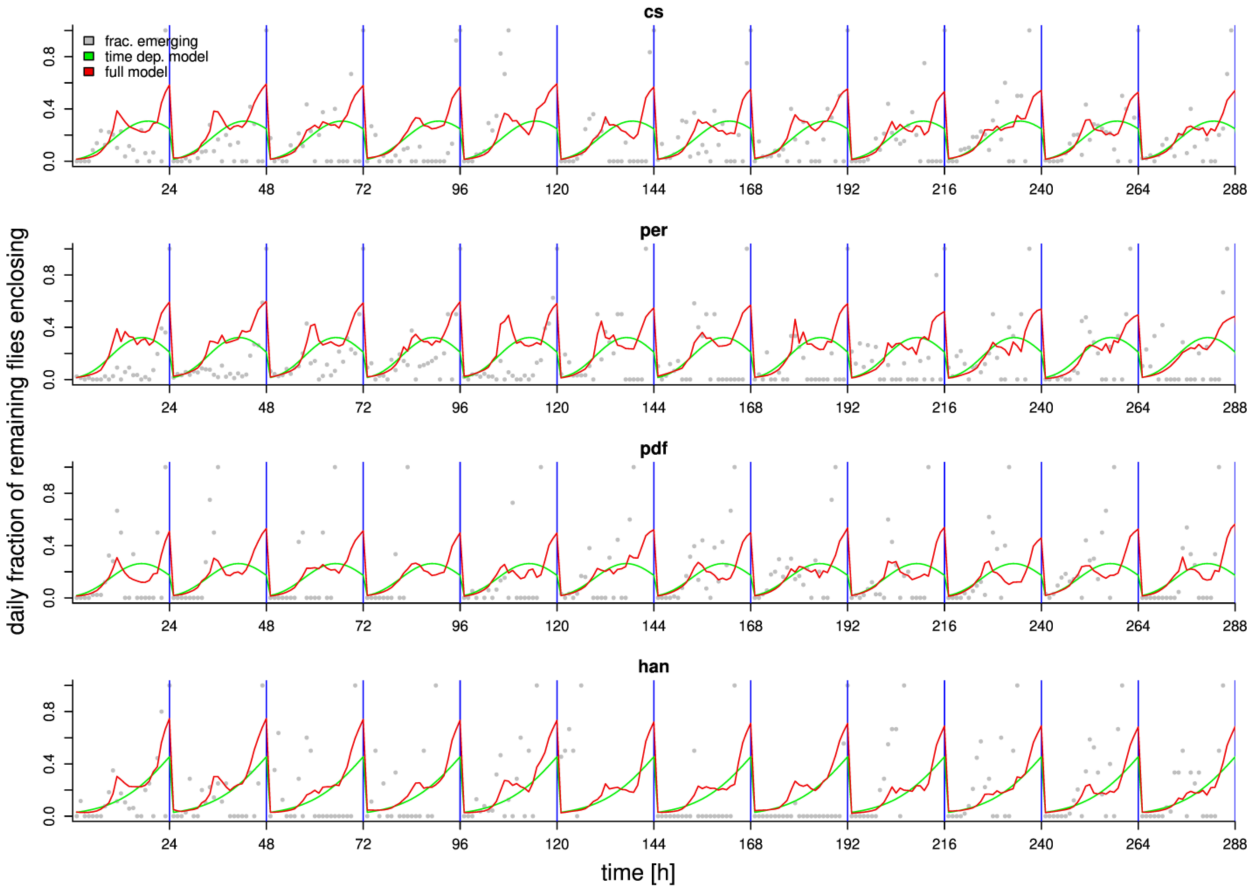

Supplementary Fig 6. Comparison of model predictions between four genotypes. Grey points show the proportion of flies eclosing during each hour that did not emerge up to this moment during that day. Note that the last empirical observation during the day must thus take the value 1 when the last flies of the day emerge. The green line shows the predicted emergence based on hour and hour^2 only whereas the red line shows the curve for the best fitting model also accounting for the effects of temperature, light and time of sun-rise (dawn). For clarity the figure shows only data and model for 12 days.
